# An educational initiative aimed at increasing antimicrobial resistance awareness among school-going Jordanian youth

**DOI:** 10.3389/fpubh.2024.1462976

**Published:** 2024-12-12

**Authors:** Basmah Qenab, Tahreer Aqel, Hanin Younis, Rahmeh AbuShweimeh, Anwaar Al Zghoul, Zaina Sweedan, Mohammad Omran, Mohan P. Joshi, Niranjan Konduri

**Affiliations:** ^1^USAID Medicines, Technologies, and Pharmaceutical Services Program, Management Sciences for Health, Amman, Jordan; ^2^USAID Medicines, Technologies, and Pharmaceutical Services Program, Management Sciences for Health, Arlington, VA, United States

**Keywords:** antimicrobial resistance, antibiotic resistance, antibiotics, education, awareness, school, children, Jordan

## Abstract

**Background:**

The World Health Organization (WHO) has recommended strategies and actions to enhance awareness and understanding of AMR. Gaps in AMR awareness remain in Jordan, particularly among the youth.

**Aim:**

To describe our programmatic approach to AMR education across Jordanian governorates among school-aged children.

**Methods:**

Our approach depicts the development of comprehensive health messages, pilot and expansion phases in schools, and pre- and post-session knowledge assessments.

**Results:**

2,700 students across 30 schools reached with AMR health messages. Gained knowledge was assessed in 932 students, revealing significant improvements in understanding the importance of consulting a doctor before taking antibiotics, the relationship between nutrition and immunity, the dangers of sharing medicines, and proper handwashing techniques. The average expenditure was approximately $8.55 per student.

**Conclusion:**

The intervention underscores the critical role of targeted educational initiatives in improving AMR awareness among youth, emphasizing the need for sustained and scalable approaches to combat AMR effectively.

## Introduction

Antimicrobial resistance (AMR) is a critical global health threat that contributed to an estimated 1.27 million deaths in 2019 (1). If not properly contained, this global problem will cause as many as 10 million deaths a year (2). In 2015, the World Health Organization (WHO) released a Global Action Plan on AMR, recommending all its member states to develop and implement country-specific action plans. With extensive national consultation and assistance from different international development partners, Jordan developed its National Action Plan for 2018–2022 (3) which was later updated for 2023–2025 (4). This National Action Plan covers all five broad objectives enunciated in the WHO Global Action Plan, including targeted awareness and behavioral modification campaigns carried out through public communication programs that aim to reach diverse demographics. Since the rollout of this action plan, WHO has been convening the World AMR Awareness Week (WAAW) (5), which is conducted annually to improve AMR awareness and understanding globally and in Jordan (6). Despite these efforts, several studies conducted from 2019 to 2021 reported a prevalent gap in awareness and understanding of AMR persisting within Jordanian communities that often leads to various forms of inappropriate antimicrobial use, including self-medication, taking incomplete courses, and the sharing of antibiotics (7, 8). A comprehensive cross-sectional survey of 620 households in Amman, Jordan’s capital, underscored the disconcerting deficiency in knowledge and awareness regarding the appropriate use of antibiotics and the nuanced implications of AMR (9). The Tracking AMR Self-Assessment Survey conducted in Jordan in 2022 reported an absence of nationwide AMR awareness campaigns and youth education on AMR (10). In this quadripartite report, the education on AMR for school-going children and youth is recommended as an intervention to improve AMR awareness in communities.

The demographic group of individuals 10 to 24 years of age constitutes a substantial portion (almost 30%) of Jordan’s population (11, 12). The lack of awareness of AMR within this segment poses a significant concern, given its pivotal influence on future health practices (13). Educating young individuals on AMR would ultimately result in well-informed adults who can appreciate the risk of increased antibiotic resistance (14). A survey in the United Kingdom reported that youth under 25 years of age are four times more likely to take antibiotics that were not prescribed for them than people who are 25 years or older (15). Targeting young people also brings the advantage of potentially reaching their family members, as the messages can be taken home from school and passed on to others in their households.

This paper describes our programmatic approach implemented across all Jordanian governorates with an objective of increasing awareness and knowledge on AMR among school-going Jordanian youth.

## Materials and methods

### Stakeholder engagement for formulating comprehensive health messages on AMR

The Jordanian Ministry of Health (MOH) led coordination meetings with key stakeholders, including the School Health Directorate and the Health Communication and Awareness Directorate, to develop comprehensive health messages on AMR and infection prevention and control (IPC). Stakeholders deliberated on a variety of priority topics (Table 1).

**Table 1:** AMR and IPC health messages topics.

AMR health messages	IPC health messages
Overview of microbes and their types	Role of a balanced diet and exercise in boosting immunity
Overview of antimicrobials and their types	Vaccination for preventing infections Environmental health and AMR
Occurrence of AMR	
Effect of AMR on individuals and communities	Improving hygiene and IPC (masking, distancing, hand washing, and hand hygiene demonstrations)
Ways to prevent AMR	
Safe use of antimicrobials	

During the health messages development process, the MOH utilized communication materials on AMR that were available on the e-Bug website (16) and material resources from the WHO (17). Subsequently, the health messages were transformed into display posters, pamphlets, bookmarks and photo frames for use during the awareness sessions (supplementary file).

### Pilot phase in select schools, followed by expansion to additional schools

The MOH selected the top three Jordanian governorates with the highest student density in the country—Amman, Irbid, and Zarqa—for the initial pilot phase. In these governorates, the Ministry of Education nominated eight secondary schools for boys and girls (two private and six public) to participate in the piloting phase. Additionally, eight focal points at the Health Affairs Directorates who perform school health education in the selected governates as part of their job description were nominated by the MOH. These eight health educators received specialized training on strategies for delivering tailored AMR and IPC health messages to students. Subsequently, the health educators conducted a series of in-person awareness sessions within the eight nominated schools. The MOH requested approximately 150 students per session.

Building on the success achieved in the pilot phase, the MOH replicated the comprehensive training program, refining it with lessons learned and feedback from the health educators. One of the challenges highlighted in the feedback was the difficulty in controlling and evaluating session impact. Due to this challenge, the MOH reduced the number of students required per session from 150 to 75. The MOH then provided this refined program to 22 newly nominated health educators for roll out to more schools. The health educators expanded the initiative to 22 additional schools (all public secondary schools for boys and girls) across the northern and southern regions of Jordan.

### Conducting the AMR and IPC awareness sessions

In both the pilot and expansion phases of implementation, secondary school students aged 14–18 years were targeted for participation in the AMR and IPC in-person awareness sessions. The sessions were crafted to cultivate an environment conducive to engagement and active participation. A variety of tools were used by the trained health educators to impart critical concepts to the students, including an animated video (18), multimedia presentation, interactive activities, group discussions, and real-world case studies. These multimodal tools were tailored to resonate with the diverse learning styles and preferences of the students, ensuring an inclusive and comprehensive educational experience. The integration of open discussion and thought-provoking inquiries related to antibiotic use and resistance was also central to these sessions. The participatory approach taken in the sessions aimed to prompt reflection and foster a deeper understanding of the subject matter among students. Table 2 summarizes the activities conducted during each session. The participation pool in the sessions included students who voluntarily engaged in the awareness sessions, signifying their interest and commitment to learning about antibiotic use and resistance. Despite efforts to engage all students aged 14–18 years at each of the selected schools, some opted not to participate due to conflicting academic obligations.

**Table 2:** Awareness session activities and their descriptions.

Activity	Description
Animated video	An engaging animated video was created to illustrate the story of a young boy whose parents habitually give him antibiotics from the cabinet whenever he falls ill. The narrative takes a turn when the boy becomes gravely sick and, alarmingly, the doctors find themselves unable to treat him effectively. This video serves as a compelling educational tool to explain the development of AMR in such scenarios. A health educator presents this video to students, initiating a discussion to explore their perspectives, subjective experiences, and reflections on the video. This activity aims to foster a deeper understanding of AMR and its impact on the individual, household, and community public health.
Multimedia presentation	Dynamic multimedia presentation was employed to deliver comprehensive information on AMR and IPC, incorporating visual aids and interactive elements. This presentation covered diverse topics, including the mechanisms of AMR, its global impact, and strategies for infection control in community and health care settings. The presentation also highlighted these AMR-related topics, such as the occurrence and impact of AMR on individuals and communities, the role of a balanced diet and exercise in boosting immunity, the importance of vaccination in preventing infections, and the link between environmental health, food safety and AMR.
Group discussions	Group discussions provided a platform for students to exchange ideas, share perspectives, and deepen their understanding of AMR and IPC by interacting with their peers and sharing experiences from their life. Health educators encouraged collaborative learning and critical thinking by posing thought-provoking questions and guiding discussions on ethical dilemmas related to antibiotic use.
Real-world case studies	Real-world case studies were presented to illustrate the practical implications of AMR and IPC, facilitating contextualized learning and critical thinking. Students analyzed case scenarios depicting antibiotic misuse, health care-associated infections, and outbreaks, and discussed potential solutions and preventive measures to address these challenges.
Role-playing exercises	Role-playing exercises allowed students to enact scenarios related to AMR and IPC, assuming distinct roles such as health care providers, patients, and policymakers. Through role-play, students gained insights into the complexities of decision making and communication in real-life settings while also exploring the consequences of antibiotic misuse and the importance of collaborative efforts in combating AMR. These role plays also made learning a fun experience.

### Pre- and post-session knowledge assessments and identifying misconceptions about AMR

During each session, the health educator conducted a pre-session knowledge assessment to gauge students’ initial understanding and reasoning about AMR. This involved presenting four closed-ended questions (Table 3) on a screen and asking students to indicate their answers by raising their hands. The health educators then tallied the correct and incorrect responses for each question. This process was repeated after the sessions to capture any shift in students’ knowledge, thereby allowing for a comparison of initial perceptions versus evolved viewpoints post-session.

**Table 3:** Students’ knowledge assessment on antibiotic use.

Question	n = 932		*X^2^*	*P*
	% (n) Pre-session	% (n) Post-session		
Correctly answered the question “Is it acceptable to take antibiotics and medication without consulting a doctor?”	53 (492)	91 (852)	96.429	<0.0001*
Correctly answered the question “Is there a relationship between proper nutrition and body immunity?”	72 (669)	86 (805)	12.548	0.0004*
Correctly answered the question “If my sibling/friend and I have a high fever and sore throat, is it okay to share the same medicine bottle or capsule strip?”	59 (550)	86 (805)	47.989	<0.0001*
Correctly answered the question “Is there a recommended “standard way” to wash hands?”	49 (458)	85 (794)	90.173	<0.0001*

*p < 0.05 Significance after test correction.

Following the initial assessment, educators posed additional open-ended questions based on the students’ responses. This interactive element encouraged students to express their thoughts and understanding of antibiotic use, resistance, and related behaviors. Topics covered included antibiotic sharing practices, understanding of proper hand-washing techniques, and other areas related to antibiotic use. These questions were informed by similar studies published in the literature (19–22).

The awareness session’s impact was assessed through a chi-square (*χ*^2^) test for independence, comparing correct responses to the four questions before and after the sessions. Additionally, a *Z*-test for proportions was used to compare the accuracy of answers pre- and post-session. Statistical analyses were conducted using RStudio, with a *p*-value of <0.05 deemed statistically significant. The Bonferroni method was applied for test correction (23).

Concurrently, health educators conducted naturalistic observations (24) during the awareness sessions’ open discussions to identify prevalent misconceptions about AMR among students. To facilitate these discussions, we drew upon existing research on AMR-related misconceptions (7–9, 25), which informed the topics and questions raised during these interactive segments.

## Results

**Figure 1:**
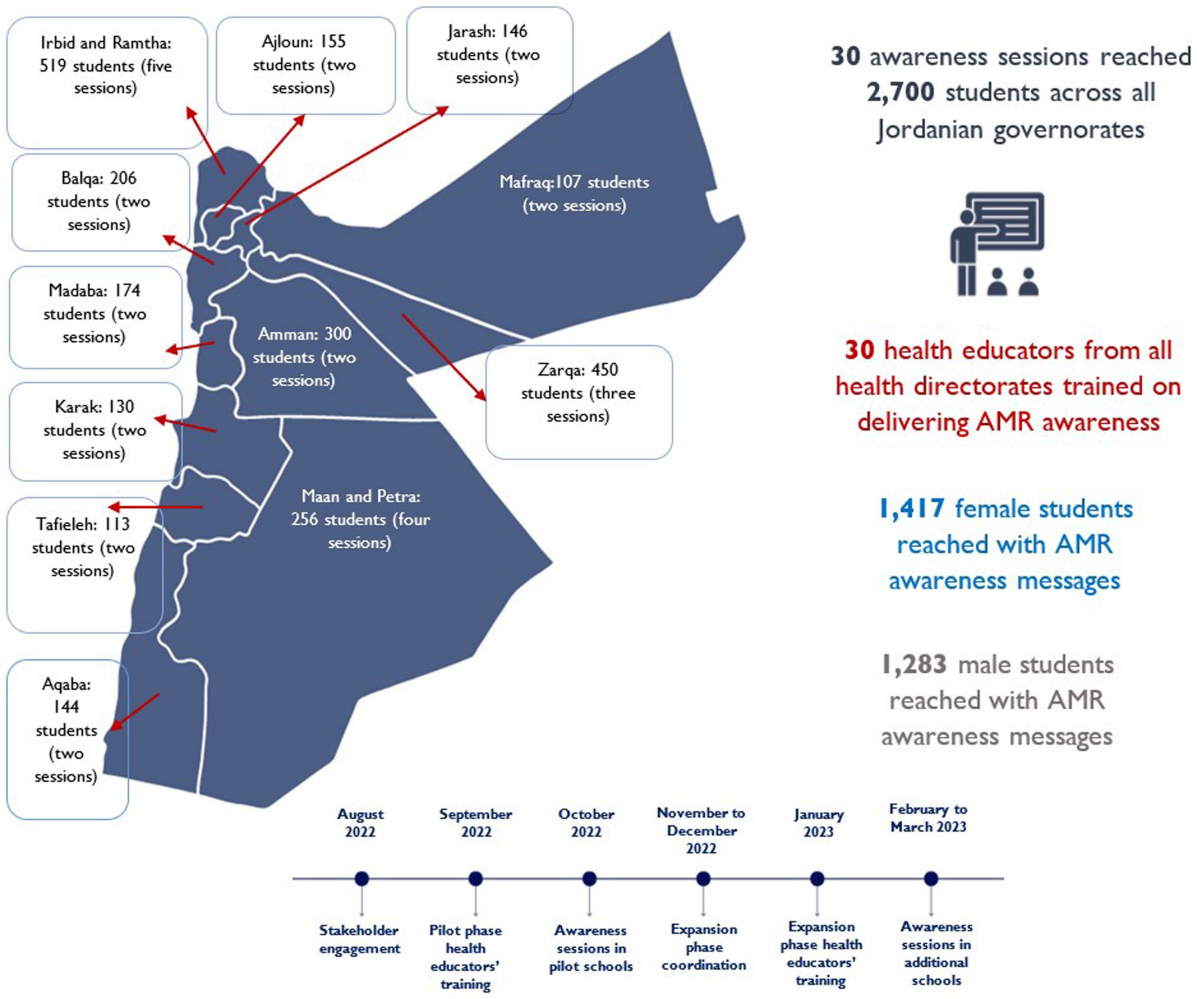
This map details the spread of educational sessions across Jordan’s governorates, marking the number of sessions and students. It also highlights the total engagement, showcasing both the cumulative number of health educators and students, and the implementation timeline.

The awareness sessions successfully reached a total of 2,700 school students aged 14–18 years from 30 secondary schools (2 private and 28 public) for boys and girls across all Jordanian governorates (Figure 1). The pilot phase awareness sessions extended over 1 month, taking place in October 2022, while the expansion phase spanned 2 months, occurring in February and March 2023. In the pilot phase, 1,125 students were reached from 8 schools (2 private and 6 public), whereas in the expansion phase, 1,575 students were reached from 22 schools, all of which were public.

The health educators were only able to collect the pre- and post-session knowledge assessment in schools where the average attendance per session was around 60 students. Consequently, the assessment was gathered from 932 students across 15 schools during the expansion phase. Among the 932 students, while 53% believed taking antibiotics without consulting a doctor was unacceptable prior to the session on the subject, that number increased to 91% after the session. Similarly, awareness about the relationship between nutrition and immunity rose from 72 to 86%. With respect to the inappropriateness of sharing medicines with others, understanding among the students of the correct behavior stood at 59% initially but increased to 86% post-session. Lastly, knowledge about proper handwashing improved from 49 to 85%. A comparison of the correct answers given before and after the sessions revealed a significant increase in understanding across these topics (Table 3).

Furthermore, misconceptions regarding AMR that were mentioned by students during the open discussions (Table 4) were corrected by the health educators. These included the notions that antibiotics can treat viral infections, antibiotics can be shared among individuals, and completing a full course of antibiotics is unnecessary once symptoms improve.

**Table 4:** Common misconceptions about antimicrobial resistance mentioned by school students during the awareness sessions.

Antibiotics are effective against viral infections, such as the common cold or flu.
Antibiotics can be shared among family members or friends to treat similar symptoms.
Completing a full course of antibiotics is unnecessary once symptoms improve.
Antibiotics are harmless medications with no potential side effects or risks when misused.
Antibiotics are effective for alleviating symptoms of illness, regardless of its underlying cause.
Antibiotic resistance only impacts individuals who use antibiotics (i.e., failure to recognize AMR as a broader public health concern).
Self-medication is effective as I am more familiar with my own body than the doctor.

The direct costs of delivering the awareness sessions to 2,700 students was JOD (Jordanian Dinar) 16,360 (US$23,075) for an average expenditure of approximately JOD 6.06 (US$8.55) per student (Table 5).

**Table 5:** Breakdown of direct costs for AMR awareness sessions.

Budget Item	Cost (JOD)	Cost (USD)
Participants training/workshop (catering service and lodging)	1,676.25	2,364.25
Posters, bookmarks, and pamphlets	12,137.20	17,118.80
Equipment rental for session facilitation	1,935.00	2,729.20
Car rental and transportation for the health educators	612.00	863.19
**Total cost**	**16,360.45**	**23,075.44**
**Cost per student**	**6.06**	**8.55**

## Discussion

Central to the initial success and subsequent rapid expansion of the awareness sessions was the engagement, collaboration and commitment of the key stakeholders at the MOH. This enabled the formulation and development of comprehensive health messages covering AMR and IPC topics and capacitated health educators from all Jordanian governates to effectively disseminate the tailored messages in schools. The sessions’ focus on raising awareness about antibiotic use and resistance, and IPC aligns with similar studies (21, 26–28).

This approach followed similar methods in previous research (26, 27, 29, 30) and recommendations from Jordan’s National Action Plan for AMR and the Tracking AMR Self-Assessment Survey (3, 10). The initial success of the initiative described in the paper motivated the key national stakeholders to integrate this school-based awareness intervention into the new iteration of Jordan’s National Action Plan on AMR (2023–2025) (4). It includes the following strategic intervention under Objective 1.1: “Implement health education programs in educational institutions and targeted health awareness programs for adolescents to raise awareness about antimicrobial resistance” and as part of this strategic intervention, it mentions activities such as developing educational curricula in schools about antimicrobials, and implementing awareness activities for students, teachers, and administrators in schools. With this, it has now become mandatory for health educators across all health directorates, demonstrating the national commitment to AMR education and prevention efforts targeted toward the public and the community.

Implementing the pilot phase followed by the expansion phase implementation strategy allowed for the gradual introduction and subsequent broadening of the intervention’s reach. Beginning in three select governorates and eight schools allowed refinement of the approach and materials before scaling up the initiative further. The experience gained from the pilot helped inform the steady expansion of the intervention to 22 additional schools. Our intervention’s scalability, ultimately covering 30 schools, marks a significant departure from the more localized efforts noted in studies from Portugal, Ghana, and England (21, 31, 32). Unlike those studies, which used methods such as storytelling or debates, our large-scale approach utilized uniform sessions to disseminate knowledge broadly. Interestingly, our unique and simple method of group hand-raising for knowledge assessment, though less detailed than individual questionnaires, encouraged immediate engagement, distinguishing our study from others while still achieving enhanced AMR awareness.

The insights from previous Jordanian studies on public/community awareness (7–9), coupled with observations on irrational antibiotic use in Europe (25) and our findings, turn a nuanced lens on the issue of antibiotic misuse and awareness about AMR. The disparity in degree of understanding across Jordan underscores a socioeconomic divide in health literacy that is mirrored in Europe. Despite a shift toward more prudent antibiotic use in Jordan (7), reliance on self-medication and informal medical advice remains a challenge. Our study amplifies these concerns, particularly the misuse of antibiotics for viral infections among students, pointing to a broad issue of insufficient health literacy on AMR.

A limitation of the sessions conducted was that they focused solely on AMR awareness in the human health sector (despite introducing the link between environmental health, food safety, and AMR), and thus they would not have provided a comprehensive spectrum of awareness. The lack of a broader focus on One Health principles, which integrate the interconnections between human, animal, and environmental health, limits students’ ability to fully understand that AMR is a multisectoral problem requiring multisectoral solutions. Future initiatives targeting school students should address this limitation by adequately incorporating practical One Health topics in awareness programs to help foster a more holistic understanding of AMR among young learners. Evidence suggests that incorporating One Health concepts into school programs increases students’ engagement and comprehension of complex health issues (33). Furthermore, introducing One Health topics early in education encourages students to think critically about the broader environmental and social factors affecting health (34).

Given the immense importance of One Health in our interconnected world, we also recommend a formal integration of One Health AMR topics into the Jordanian school curriculum. This approach will provide opportunity to introduce, in simple terms, the need for multisectoral and whole-of-society efforts to combat AMR, including the elimination of unnecessary use of antimicrobials in both humans and animals and preventing unregulated discharge of infectious wastes from hospitals into the environment such as rivers and soil. A study from Uganda (35) supports this approach and emphasizes the importance of enhancing schools’ curriculum to encompass a wider scope of AMR, One Health, and global health security concepts. This would foster a comprehensive understanding among students, thereby improving their awareness and actions with respect to AMR.

Furthermore, ongoing training for school health educators on AMR and IPC by the Health Affairs Directorate can ensure that those educators are well-equipped with the latest knowledge and teaching methodologies. The MOH’s collaboration with international bodies can amplify AMR awareness, leveraging digital platforms for a wider reach. Given the global threat associated with AMR, cross-border collaborations are essential for sharing successful strategies and enhancing research efforts.

Our study’s reliance on group hand-raising for assessment, while innovative, introduces potential biases and may not capture nuanced changes in individual understanding, long-term retention of knowledge or behavior change. Evidence shows that behavior change activities for students require time and follow-up to be effective, with longitudinal interventions that span several months to years and regular check-ins yielding lasting improvements (36). Continuous feedback and reinforcement, such as the Check-In Check-Out system, help students stay on track (37, 38). Engaging parents, the community (20, 39), and using digital platforms for follow-up support further enhance student outcomes (40). However, due to budget constraints, long-term follow-up was not possible, and the challenge in securing parental consent for more detailed questionnaires highlighted the need for better communication between schools and parents. Additionally, budgetary and logistical constraints limited our ability to collect and follow-up data digitally. Future studies should explore more cost-effective methods for long-term follow-up, improve strategies for securing parental consent, and incorporate digital tools to overcome these limitations and better support sustained behavior change.

The knowledge assessment did not track individual students’ progress; therefore, pre- and post-session results reflect group level comparisons rather than changes in individual responses. This approach is similar to a study (41) that collected anonymous pre and post assessment results. While this limits the ability to measure individual knowledge retention, collecting anonymous data was more feasible for this intervention due to budgetary constraints; this approach avoided the need for more complex and costly systems required for tracking individual responses digitally. Additionally, since no identifiable information was collected, the process did not require parental consent, which further simplified the data collection process.

Moreover, despite the increase in knowledge post-intervention, not all students reached a complete understanding of AMR-related topics. Achieving “complete understanding” would imply that every student answered all questions correctly. While the results show significant improvements in post-session responses, none of the students achieved a 100% correct response rate, indicating that there were still gaps in students’ understanding of some of the AMR-related topics. This can be attributed to AMR inherently being a complex topic (42), and to the variations in students’ prior knowledge and engagement levels (43).Only four questions were used in the assessment due to time constraints and the need to ensure a manageable workload for both the health educators and the students. Future studies could incorporate a more comprehensive and detailed assessment, including questions not only about AMR knowledge but also about attitudes toward antimicrobial use and One Health, allowing for a broader evaluation of student understanding and providing more opportunities for learning (19, 21).

Additionally, due to the programmatic nature of this study and logistical constraints in the school system, implementing a control group was not feasible which limited our ability to directly compare the outcomes with those of a group that did not receive the intervention. Future studies should aim to incorporate more comprehensive evaluation methods, including the use of control groups, to better assess the intervention’s true effectiveness (44).

One of the challenges encountered was the difficulty in managing and conducting the knowledge assessments in schools where the average session attendance exceeded 60 students. In the pilot phase, attendance reached around 150 students per session, making it difficult to conduct the assessments because of the class size. To address the logistical and session control challenges experienced during the pilot phase, the MOH opted to reduce the number of students per session to around 75 during the expansion phase. However, not all schools adhered strictly to this number, as attendance varied depending on students’ availability. For example, in some schools, certain students were unavailable due to scheduling conflicts or exams, resulting in sessions that averaged 60 students. These smaller student numbers allowed the health educators to manage the assessment effectively. The assessment was conducted in 15 of the 22 schools, covering 932 students. In the remaining 7 schools, more students joined the sessions, exceeding 75 participants, which made it challenging for the health educators to manage and conduct the assessment. This underscores the importance of class size in such interventions. Although explicit research on the ideal class size for health awareness sessions is limited, numerous studies support the effectiveness of small group learning (45).

This study identified a direct cost of US$8.55 per student for an AMR awareness intervention that reached 2,700 students, exclusive of indirect costs, which can be more challenging to quantify and may vary depending on the context. In alignment with our approach, a school health screening program (46) similarly reports solely on direct costs, with expenditures ranging from US$8.88 to US$13.64 for each of the 2,928 enrolled participants. Conversely, a study from Kenya (47) reported a cost of US$41.66 per person in its initial implementation for 47,133 individuals, which represents both direct and indirect expenses. Such differences in accounting for costs underscore the challenge of making direct comparisons across studies and emphasizes the necessity of tailoring intervention strategies to specific contexts. To address cost concerns, one potential approach involves leveraging existing resources within the education system. By training health or science-related teachers to deliver awareness on AMR at their own schools, the logistical burden and associated costs can be reduced. This will also support further integration and institutionalization of the intervention within the implementation setting itself and help the in-school teachers become more aware and competent regarding relevant AMR and IPC topics. Such cost-saving measures enhance the economic efficiency and longer-term logistical and financial sustainability of interventions.

Future efforts should focus on overcoming methodological limitations and fostering a more nuanced understanding of AMR across diverse populations. Additionally, it is imperative that subsequent studies delve into the cost-effectiveness of such educational interventions, particularly in relation to long-term health outcomes and behavior modification. This will enable a more thorough evaluation of their impact and value, thereby informing more strategic and effective AMR mitigation strategies.

## Conclusion

This study contributes to the growing body of evidence on effective AMR education, highlighting the necessity of scalable, interactive interventions. Comprehensive success in AMR containment cannot be achieved without adequately addressing knowledge gaps and misconceptions among the pubic and the community. School students are an easy-to-reach target group for such initiatives, and assessment of change in knowledge and awareness is also relatively easy as schools offer a contained environment.

## Data Availability

The raw data supporting the conclusions of this article will be made available by the authors, without undue reservation.
