# Supplementary material for: An educational initiative aimed at increasing antimicrobial resistance awareness among school-going Jordanian youth
Source: Front Public Health. 2024 Dec 12;12:1462976. doi: 10.3389/fpubh.2024.1462976 (PMC11669698; doi:10.3389/fpubh.2024.1462976)

**Supplementary File: Display posters, pamphlets, bookmarks and photo frames used during the awareness sessions**

**Posters**


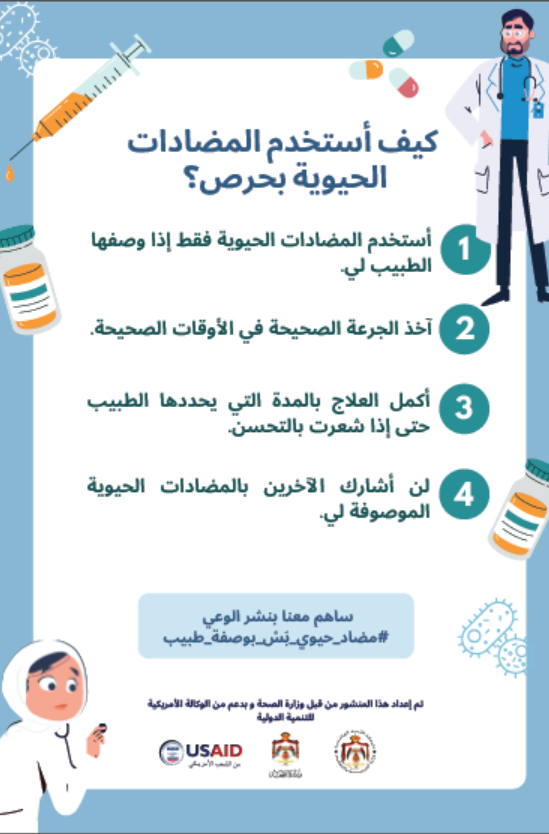


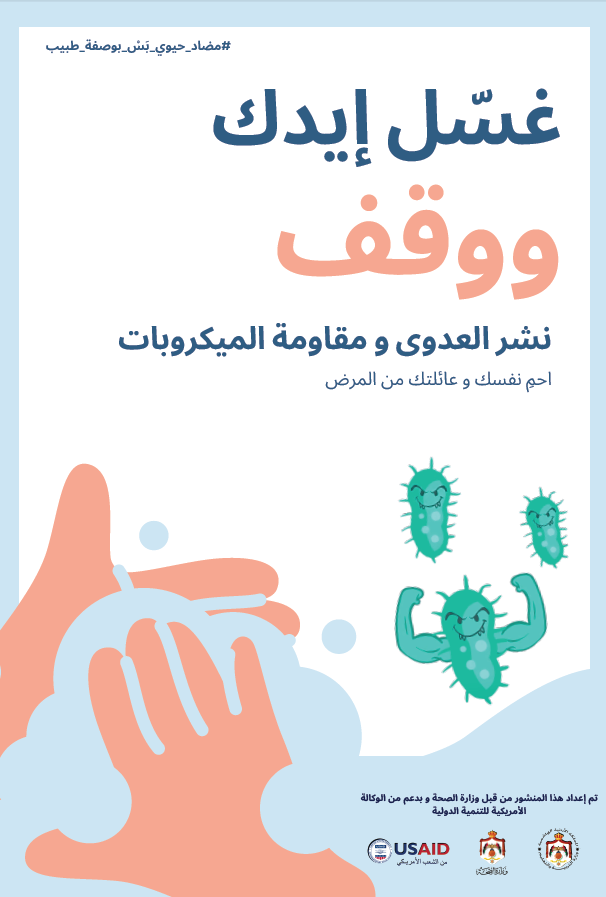


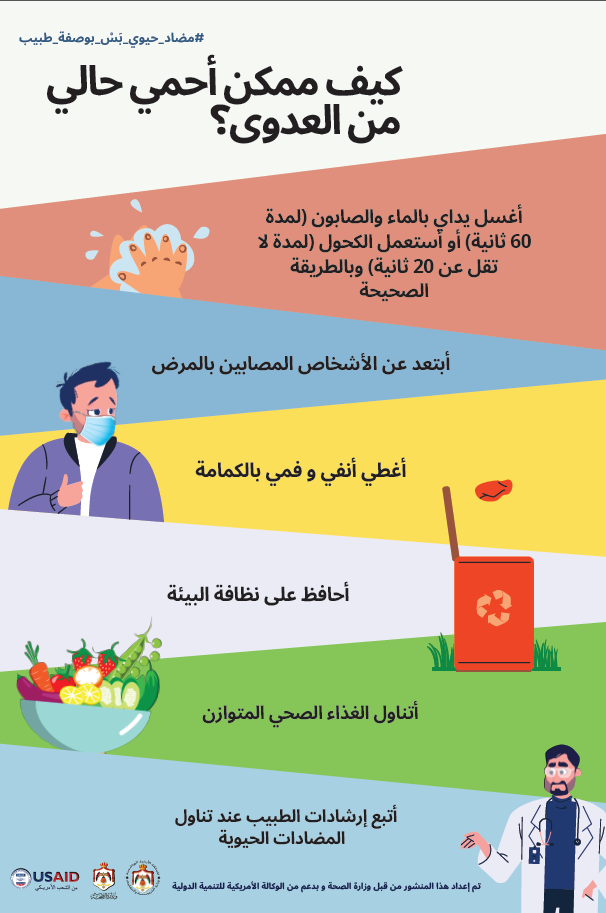


**Pamphlets**


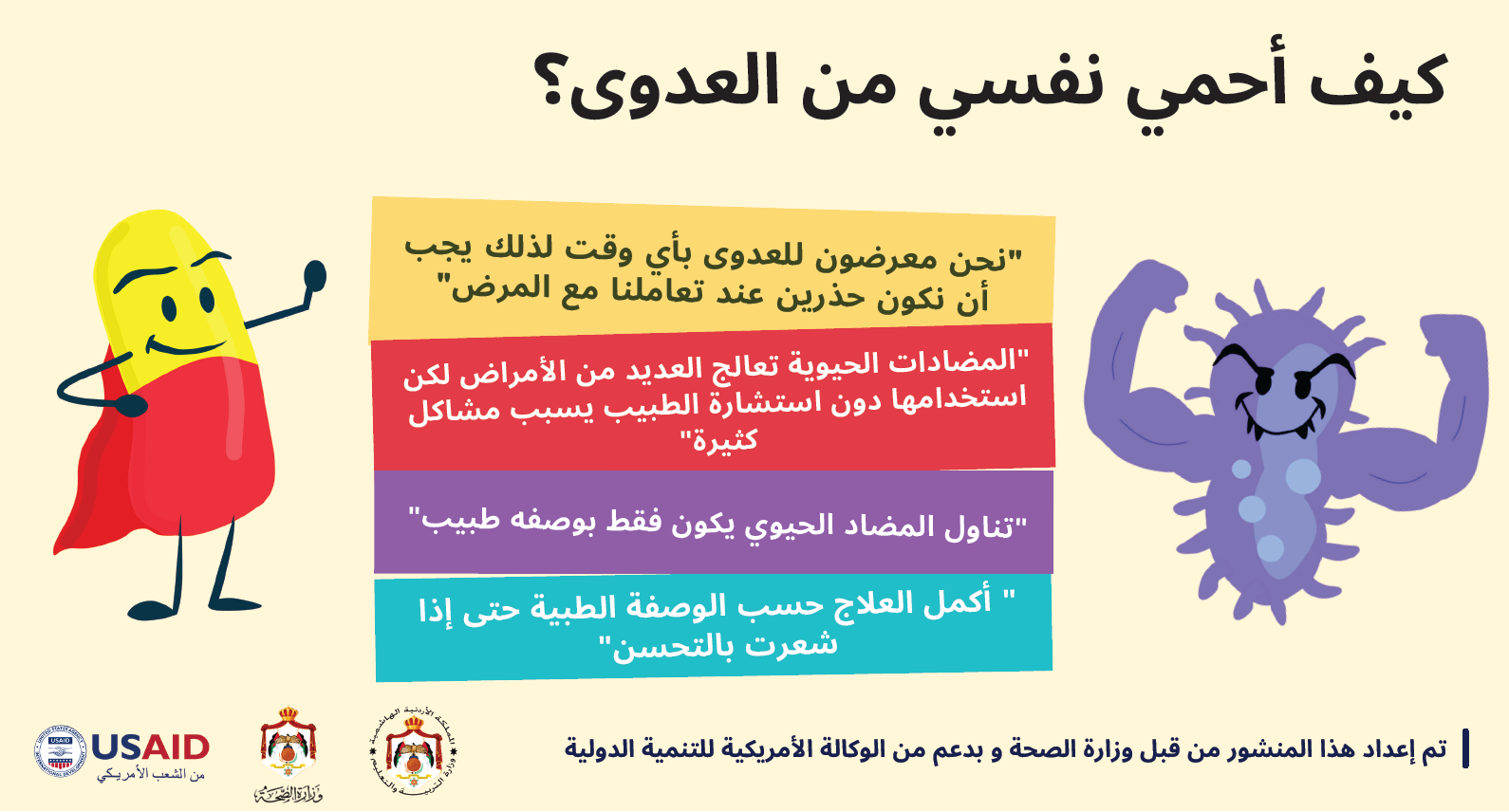


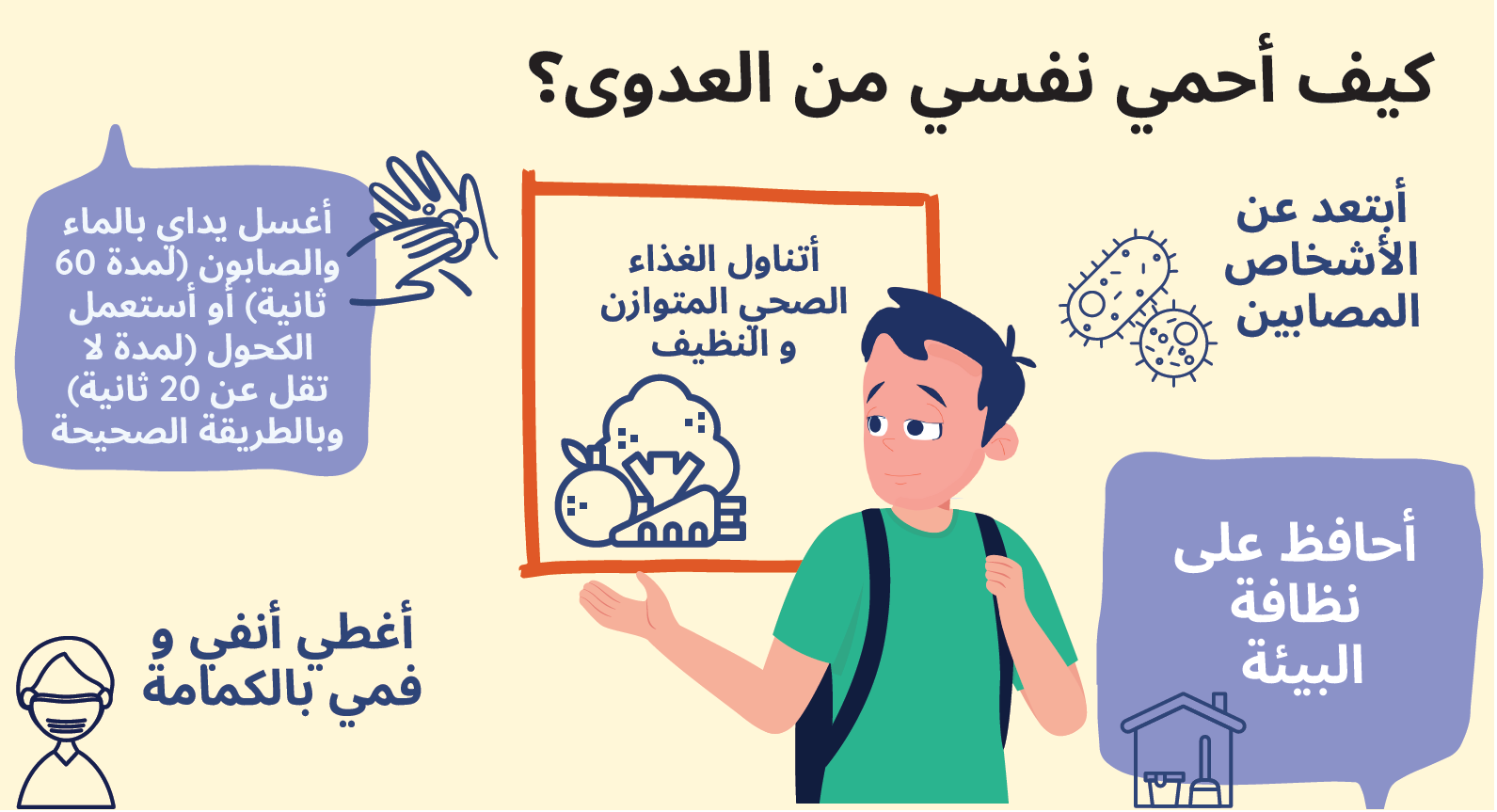


**Bookmarks**


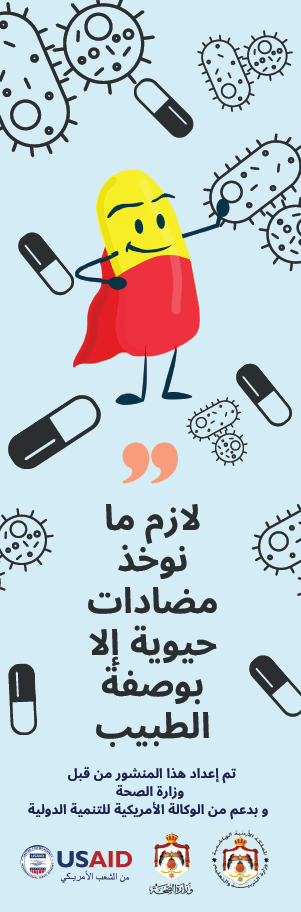


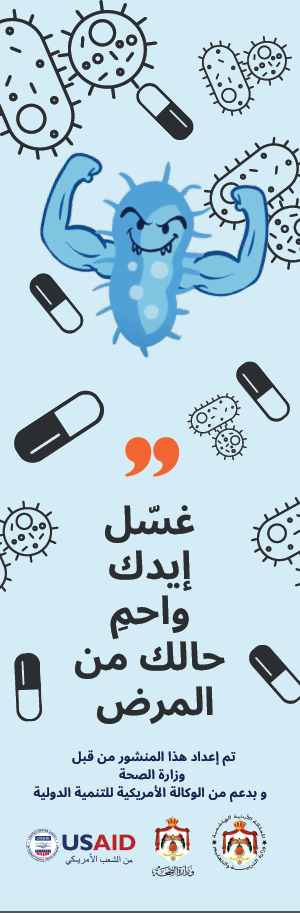


**Photo Frame**


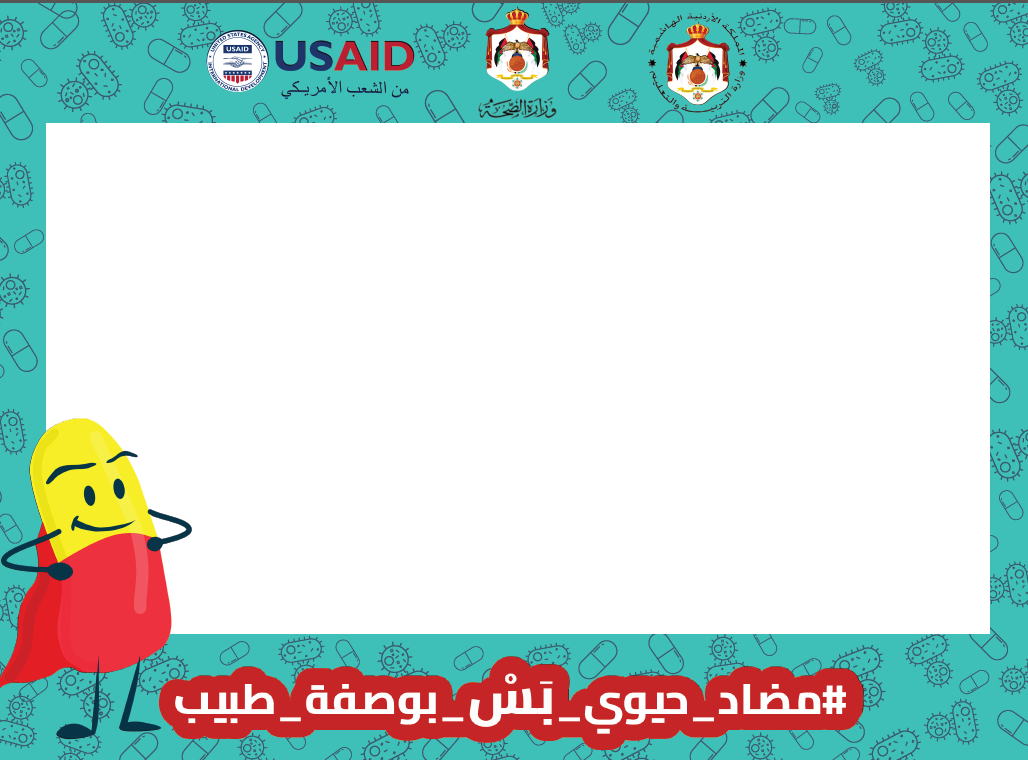


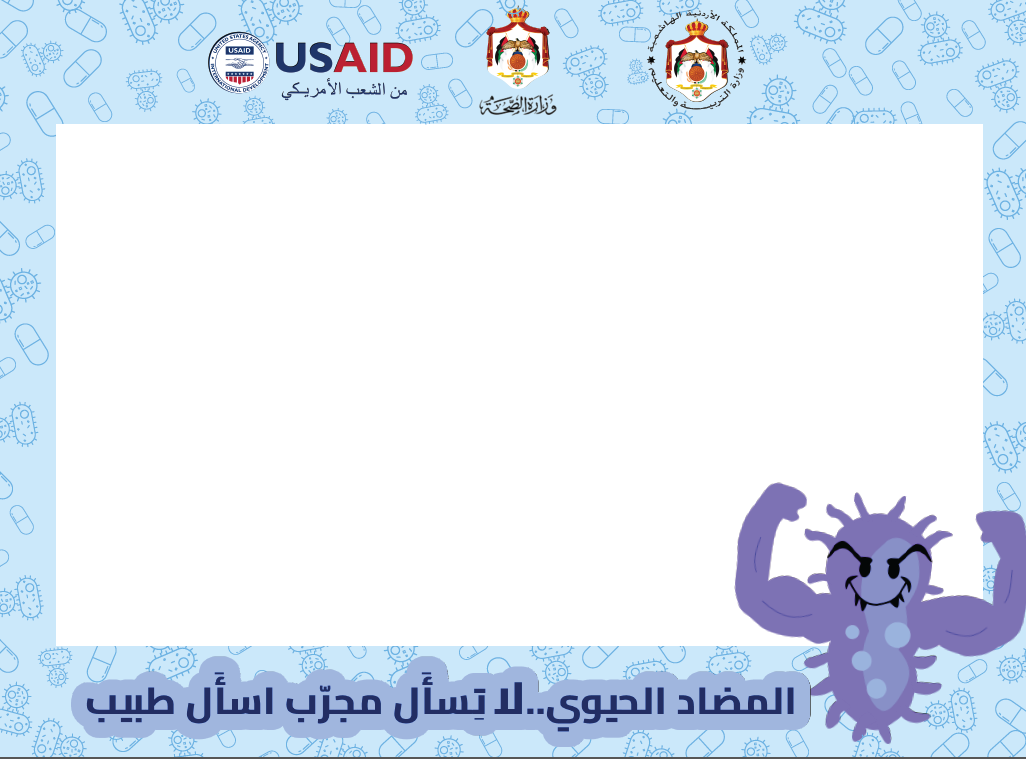

Supplement: Supplementary file 1 [file Data_Sheet_1.docx]
